# Herb-Partitioned Moxibustion and the miRNAs Related to Crohn's Disease: A Study Based on Rat Models

**DOI:** 10.1155/2015/265238

**Published:** 2015-02-25

**Authors:** Kai Wei, Dan Zhang, Jue Hong, Cuihong Zhang, Xiaoming Feng, Yan Huang, Jie Liu, Lingxiang Wu, Huangan Wu, Xiaopeng Ma

**Affiliations:** ^1^Department of Integrated Traditional Chinese and Western Medicine, Huashan Hospital, Fudan University, Shanghai 200030, China; ^2^Shanghai University of Traditional Chinese Medicine, Shanghai 201203, China; ^3^Shanghai Research Institute of Acupuncture-Moxibustion and Meridians, Shanghai 200030, China

## Abstract

Crohn's disease (CD) is a major subtype of inflammatory bowel disease (IBD). Herb-partitioned moxibustion (HPM) has been proven to be effective in treating CD by a large amount of clinical and experimental researches. MiRNAs (microRNAs) are increasingly recognized as important posttranscriptional regulators of inflammatory genes. In this study, we established experimental CD rat models and investigated the miRNAs associated with the onset of experimental CD; then, we further identified CD-related miRNAs that were regulated by HPM and explored the relationship between CD and the potential target genes of involved miRNAs. We found that miR-147 and miR-205 were significantly downregulated in colons of experimental CD rats and may be closely associated with the onset of experimental CD. HPM may extenuate inflammatory responses in colons and ameliorate colonic damages in CD via upregulating the expression of miR-147 and miR-205 and then further downregulating the expression of inflammation-related mRNAs, negatively regulating inflammatory signal pathways, and reducing the production of downstream inflammatory cytokines.

## 1. Introduction

Crohn's disease (CD) is a major subtype of inflammatory bowel disease (IBD), majorly presenting chronic inflammation that can affect any part of the gastrointestinal tract, with terminal ileum most commonly involved [1]. The pathogenesis of CD is not quite clear, and an aberrant host immune response is often thought to be one of the main pathogeneses [2]. In recent years, more and more researches have demonstrated that the interactions of genetic and environmental factors in dysregulated immune responses of intestinal mucosa play an important role in the pathogenesis of CD [3, 4]. While the regulation of inflammatory gene expression is not fully understood, miRNAs are increasingly recognized as important posttranscriptional regulators of inflammatory genes [5]. miRNAs are a class of endogenous, noncoding small RNAs, which are about 22 nucleotides in length and may play important gene-regulatory roles by pairing to the complementary sequences in the 3′-untranslated region (3′-UTR) of target mRNAs to prevent protein synthesis either by transcriptional suppression or by degradation of their target mRNAs [6–8]. Each miRNA may modulate the expression of many distinct mRNAs, and conversely, any given mRNA can be targeted by different miRNAs [9]. In humans, at least one-third of all protein-coding mRNAs are thought to be regulated by miRNAs [10]. miRNAs are implicated in many biological processes, including tissue morphogenesis, major signaling pathways, and cellular processes like apoptosis, differentiation, and proliferation [11, 12]. Dysregulation of miRNA expression has been widely involved in various human diseases such as cancers and chronic inflammatory and autoimmune diseases [13, 14]. It has been demonstrated that the expression of many miRNAs is significantly changed in colonic mucosa of CD patients [1], and some miRNAs can even help to distinguish indeterminate IBD [15, 16].

As a type of moxibustion, herb-partitioned moxibustion (HPM) has a long history of being used for various diseases for its effect of dispelling cold, warmly lubricating the inside, and improving the function of meridians. It has been proven by a large amount of clinical and experimental studies that HPM is effective in treating CD [17–21]. It can help to promote the effects of Western medicine in controlling major symptoms and complications of CD, reduce the doses and side effects of Western medicine, and thereby promote the quality of CD patients' lives.

However, no research has been performed to reveal the regulating effects of acupuncture and moxibustion on miRNAs in CD until now. Therefore, we established experimental CD rat models and investigated the miRNAs associated with the onset of experimental CD; then we further identified CD-related miRNAs that were regulated by HPM and investigated the relationship between these miRNAs and inflammation, for revealing the mechanism of HPM in treating CD at a genetic level.

## 2. Materials and Methods

### 2.1. Instruments and Reagents

NanoDrop-2000 spectrophotometer was supplied by NanoDrop Technologies (Wilmington, DE, USA); 12-Bay Hybridization System was supplied by Madison (WI, USA); Axon GenePix 4000B Microarray Scanner and GenePix Pro V6.0 were supplied by Axon Instruments (Foster City, CA); LightCycler 96 PCR cycler was supplied by Roche (Basel, Switzerland); 5% TNBS (2,4,6-trinitrobenzenesulfonic acid) was supplied from Sigma (St. Louis, MO, USA); TRIzol Reagent was supplied by Invitrogen (Life Technologies, Carlsbad, USA); miRCURY LNA Array, miRCURY Power Labeling Kit, and Wash Buffer Kit were supplied by Exiqon (Vedbaek, Denmark); microRNA qRT-PCR Sybgreen Detection Kit was supplied from bioTNT (Shanghai, China).

### 2.2. Animals

Male Sprague-Dawley rats (150 ± 10 g) were housed in Department of Laboratory Animal Science of Fudan University (Certificate number SCXK (Shanghai) 2009-0019), 5 or 6 rats per cage. Rearing conditions were a laminar flow, specific pathogen-free (SPF) atmosphere, room temperature 23°C, relative humidity 50%~70%, 12 h light/dark cycle (lights on from 7:00 am to 7:00 pm), and free access to water and rodent chow. This study was carried out in strict accordance with the guidelines provided by the National Institutes of Health for the Care and Use of Laboratory Animals. The animal protocol was approved by the Committee on the Ethics of Animal Experiments of Fudan University. All surgery was performed under sodium pentobarbital anesthesia, and all efforts were made to minimize suffering.

### 2.3. CD Model Preparation

After 3 days of adaptive feeding, our experiment began when the rats behaved normally. A total of 33 rats were randomly divided into 3 groups: a normal control (NC) group, a model control (MC) group, and an HPM group, 11 rats per group. The CD models were prepared according to Morris' method [22]. Both MC and HPM groups received enema with 5% (W/V) TNBS and 50% ethanol mixed at 2 : 1, once a week for 4 weeks. After model preparation, each group sacrificed a rat to check whether the models were successfully prepared by using HE staining. The interventions began when the model was confirmed a success.

### 2.4. Groups and Treatment

**Figure 1:**
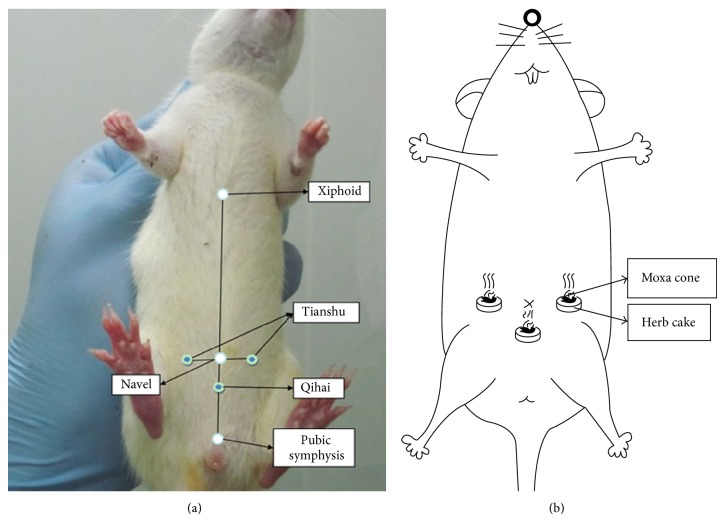
HPM treatment. (a) The location of Tianshu and Qihai; (b) operation of HPM.

HPM group: Tianshu (bilateral, ST25) and Qihai (CV6) were selected (see Figure 1(a) for the locations). The herb cakes were made of Aconiti praeparata powder and yellow wine into size of 1 cm in diameter and 0.45 cm in thickness. Moxa cones were made of about 90 mg refined moxa, of 0.6 cm in diameter and 0.6 cm high. HPM treatment was performed with the herb cakes placed on rats' Tianshu and Qihai and moxa cones placed on the herb cakes (shown in Figure 1(b)), 2 cones successively burnt for each acupoint each session, one session a day for totally 7 days.

MC and NC groups only received the same grasping and fixing as the HPM group.

### 2.5. Sampling

After treatment, all rats were sacrificed and the distal colons (6~8 cm) were harvested. Every colon was cut into 2 parts, one of which was snap frozen in liquid nitrogen and then reserved at −80°C, while the other was fixed in 10% neutral-buffered formalin.

### 2.6. Indexes Detection

#### 2.6.1. Macroscopical Score of Colonic Lesions

The distal colons were cut longitudinally along the mesentery after being harvested and then observed under a magnifier for detecting the damage of the mucosa. The general damage of rats' colons was assessed and scored according to the Score Criteria of Colonic General Damage [22, 23].

#### 2.6.2. Histopathological Observation of Colons

Formalin-fixed colons were dehydrated, embedded in paraffin, sliced at 4 *μ*m, and stained with hematoxylin-eosin (HE) to assess the inflammation and histological damage of rats' colons under optical microscope. The histological scores were assessed according to the Score Criteria of Colonic Histological Damage [24].

#### 2.6.3. miRNA Screening and Verification

(*1) RNA Extraction*. Total RNA including miRNA from the colon samples was extracted using Trizol method. Briefly, 1 mL Trizol reagent and 100 mg colon sample were put together into a 2 mL microtube and homogenized. Then add 0.2 mL chloroform and shake tubes vigorously for 15 s and incubate them at room temperature for 5 min. Centrifuge them at 4°C, 12000 rpm for 15 min, and transfer 400 *μ*L upper, aqueous phase into a new collection tube. Add 400 *μ*L precooled isopropanol and mix thoroughly by pipetting up and down several times. Incubate them on ice for 15–20 min, and then centrifuge at 4°C, 12000 rpm for 10 min. Discard the supernatant and wash precipitations twice with precooled 75% DEPC ethanol (DEPC water: ethanol = 1 : 3) and then centrifuge at 4°C, 7500 rpm for 8 min. Discard the supernatant and leave the precipitations (RNA) in air to dry. Use 20 *μ*L DEPC water to dissolve RNA. Then RNA integrity was determined by gel electrophoresis. Quality and quantity of RNA were measured by using a NanoDrop-2000 spectrophotometer. The extracted RNA samples were stored at −80°C until used.

(*2) miRNA Microarray Hybridization and Data Analysis*. To find specifically expressed miRNA(s) for CD rats' colons and target miRNA(s) of HPM, the isolated RNA samples were subjected to comprehensive analysis of miRNA expression patterns with the microarray-based technology, using the 7th generation of miRCURY LNA Array version 18.0. The experimental procedures were as follows: 1 *μ*g of each total RNA was treated with calf intestine phosphatase (CIP) buffer and CIP enzyme, denatured using DMSO, and then directly labeled with Hy3 fluorescent label using T4 RNA ligase at the 3′ end. The mixture of labeled RNA samples and hybridization buffer were first denatured and then hybridized with the miRCURY LNA Array in a 12-Bay Hybridization Systems in accordance with array manual. Following hybridization, slices were washed several times using the wash buffer kit supplied, dried by centrifugation, and finally scanned using the Axon GenePix 4000B microarray scanner. Scanned images were then imported into GenePix Pro 6.0 software for grid alignment and data extraction. Replicated miRNAs were averaged and miRNAs whose intensities ≥30 in all samples were chosen for calculating normalization factor. Expressed data were normalized using the Median normalization. All microarray data have been deposited in NCBI's Gene Expression Omnibus (GEO) and are accessible through GEO Series accession number GSE65490 (http://www.ncbi.nlm.nih.gov/geo/query/acc.cgi?acc=GSE65490) [25]. After normalization, the differences in miRNA expression were considered significant if (1) fold change >2.0 and (2) analysis of variance or nonparametric test, *P* value <0.05. Then, hierarchical clustering was performed using MEV software in the differentially expressed miRNAs from the microarray results. 

(*3) Quantitative Reverse Transcription Polymerase Chain Reaction (qRT-PCR).* Expression levels of miRNAs that showed significant changes based on the microarray results were further verified by qRT-PCR. The reverse transcription (RT) systems were formulated as follows: 1 *μ*L total RNA, 1 *μ*L 10x stem-loop RT primer, 2 *μ*L 5x RT buffer, 1 *μ*L 10 mM dNTPmix, 0.4 *μ*L efficient reverse transcriptase, and 0.25 *μ*L recombinant RNA inhibitor; add ddH_2_O to 10 *μ*L and then incubate at 42°C for 60 min and 95°C for 5 min to complete RT. After RT, cDNA should be held at −80°C if not used immediately. All PCR reaction systems were 20 *μ*L, including 1 *μ*L cDNA sample, 10 *μ*L fluorescent dyes, 2 *μ*L 10x forward primer, 2 *μ*L 10x reverse primer, and 5 *μ*L ddH_2_O. Each cDNA sample was performed in duplicate and amplified on the LightCycler 96 Real-Time PCR system as follows: (1) denatured at 95°C for 5 min; (2) 40 cycles of 95°C 10 s, 60°C 30 s, and 72°C 10 s; (3) 95°C 10 s, 65°C 60 s, and 97°C 1 s. The cycle passing threshold (Ct) was recorded for each miRNA, and the ubiquitously expressed small nuclear RNA U6B was chosen as the endogenous control for data normalization. The relative expression levels of miRNAs were calculated using 2^−ΔΔCt  ^ method.

For the RT and PCR amplification, the following rat-specific primers were used (listed in Tables 1 and 2).

### 2.7. Prediction of miRNA Target genes

The predicted target genes were inquired by the online software TargetScan release 6.2 (http://www.targetscan.org/). The predicted genes were sorted based on the context scores. Genes giving absolute values of context scores of more than 0.20 were pooled as target candidates.

### 2.8. Statistical Analysis

Statistical analysis was performed using the SPSS 15.0 software. Statistical description: data compliant with normality were presented as means ± SEM; if not, Median (min~max) was used. Statistical inference: one-way analysis of variance (one-way ANOVA) was performed if the data followed a normal distribution and variances were homogeneous; then least significant difference (LSD) test was used for further pairwise comparison; the nonparametric test was adopted if the data did not follow a normal distribution or variances were not homogeneous; then Games-Howell test was used for further pairwise comparison. Hierarchical clustering was processed by using MEV software for the differentially expressed miRNAs from the microarray results. *P* values <0.05 indicated statistical significance.

## 3. Results

### 3.1. General Damage of Rats' Colons

**Figure 2:**
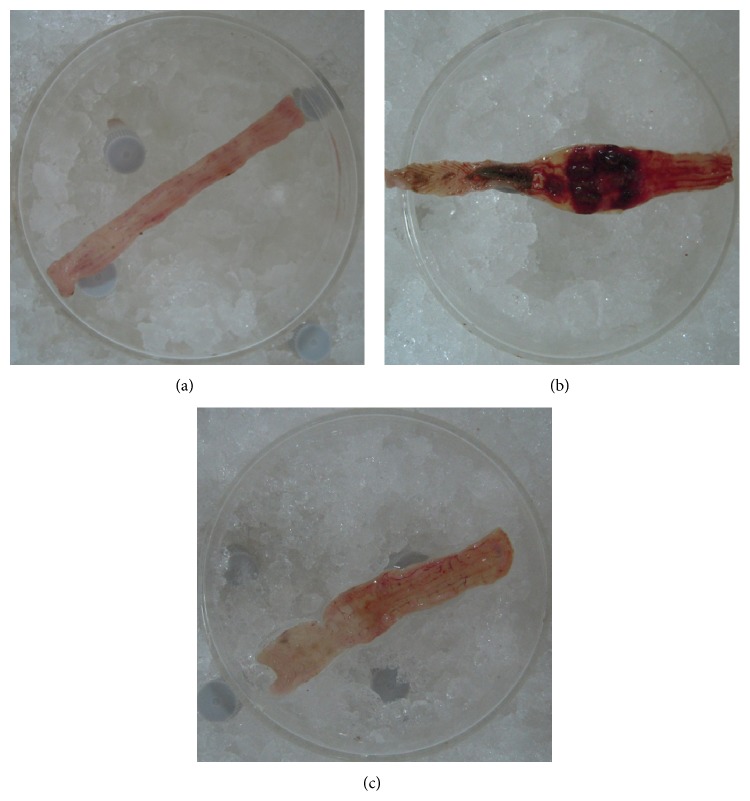
Observation of general damage of rats' colons. (a) Normal colon of NC group, with smooth intestinal mucosa; (b) colon of MC group, with severe hyperemia and edema, intestinal expansion, and ulcer; (c) colon of HPM group, with relatively smooth intestinal mucosa, mild hyperemia, and edema.

As shown in Figure 2, observed by naked eyes, colonic mucosa of the NC rats was smooth, without intestinal wall thickening, congestion, or ulcers. Colonic mucosa of the MC rats was missing, with ulcer, intestinal expansion, severe intestinal wall thickening and hyperemia, and the middle segment presenting the most serious lesions. Colonic mucosa of the HPM rats was relatively smooth, with mild congestion, edema, but without ulcer.

### 3.2. Histopathological Damage of Rats' Colons

**Figure 3:**
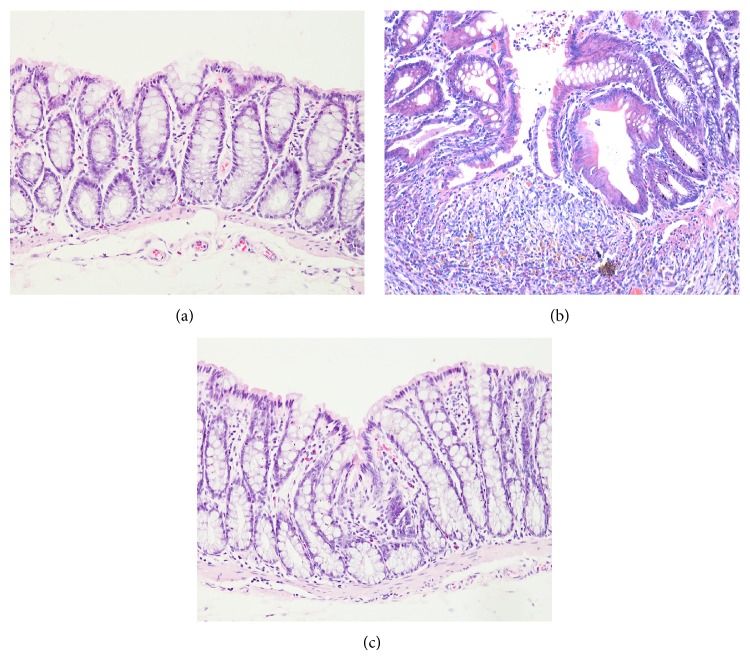
Histopathologic observation of intestinal mucosa after HPM treatment. Distal colons were taken and fixed in 10% neutral-buffered formalin; then sections were sliced at 4 *μ*m and stained with HE (×200). (a) Normal intestinal mucosa with intact structure and neatly arranged glands. (b) Remarkable fissure-like ulcer, obvious inflammatory cells infiltration, lamina propria and submucosal edema, and submucosal fibrous tissue proliferation were observed in colon of MC group. (c) Reduced inflammatory cells, slight edema in colonic mucosa and submucosal connective tissue, and integrity of mucosal epithelium were observed in colon of HPM group.

**Figure 4:**
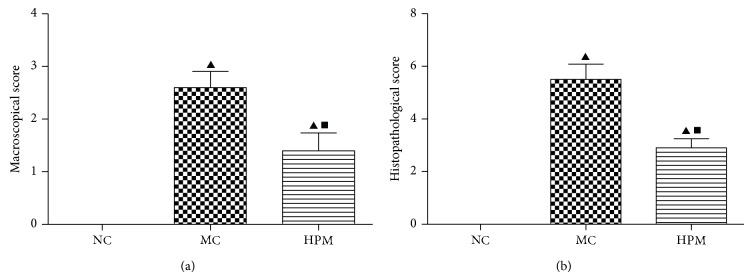
Damage scores of rats' colons. The damage scores were presented as means ± SEM, *n* = 10 rats per group. ^▲^ *P* < 0.01 versus the NC group; ^■^ *P* < 0.01 versus the MC group.

To determine the inhibitory effects of HPM on the intestinal damage of CD rats' colons, the samples were observed under light microscope after HE staining. The normal colons had structural integrity and the epithelium was continuously covered, with glands neatly arranged. As compared with the NC group, remarkable fissure-like ulcer, lack of local mucosal epithelium, obvious inflammatory cells infiltration, glands derangement with vacuolization, lamina propria and submucosal edema with many lymphocytes and eosinophils infiltration, and occasional sarcoidosis granuloma with fibrous tissue proliferation were observed in colons of the MC group, indicating successful preparation of CD model. HPM treatment significantly attenuated mucosal damage and intestinal inflammation in colons of CD rats, with the reduction of inflammatory cells infiltration, integrity of mucosal epithelium, and slight edema in colonic mucosa and submucosal connective tissue (Figure 3). The histopathological scores of the MC and HPM groups were significantly higher than that of the NC group (*P* < 0.01), and HPM could significantly decrease the histopathological scores of the CD rats' colons (*P* < 0.01) (Figure 4). Our research indicated that HPM could ameliorate intestinal inflammation and tissue morphology in colons of CD rats.

### 3.3. Differentially Expressed miRNAs in CD Rats' Colons

**Figure 5:**
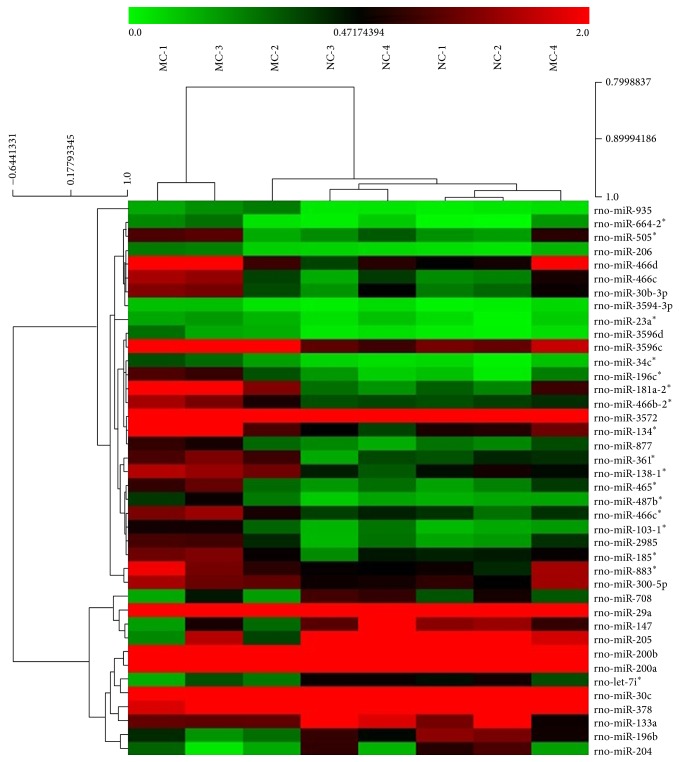
Differentially expressed miRNAs in the MC group. Forty differentially expressed miRNAs were identified by miRNA microarray in the MC group (compared to the NC group). Red indicates higher than the mean intensity (the black zone) across all samples, and green represents lower than the mean intensity. *n* = 4 rats per group.

**Figure 6:**
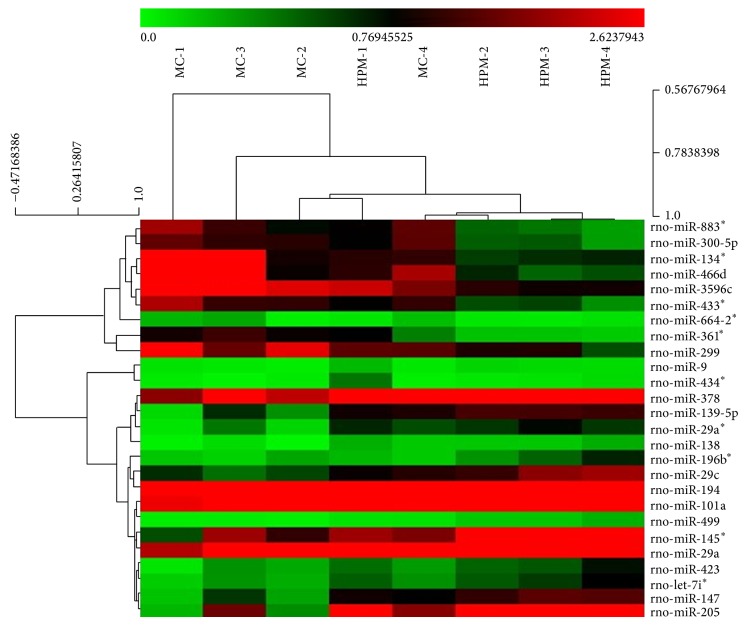
Differentially expressed miRNAs in the HPM group. Twenty-six differentially expressed miRNAs were identified by miRNA microarray in the HPM group (compared to the MC group). Red indicates higher than the mean intensity across all samples, and green represents lower than the mean intensity. *n* = 4 rats per group.

This miRCURY LNA contains 3100 capture probes and covers all human, mouse, and rat miRNAs annotated in miRBase 18.0, as well as all viral microRNAs related to these species. The expression of miRNAs in rats' colons was tested based on the miRNA microarray analysis, and the results were further analyzed using SPSS software. We found that 40 miRNAs in CD rats' colons were significantly differentially expressed compared with the normal ones (*P* < 0.05), with 28 significantly (>2-fold) overexpressed and 12 significantly downregulated (Figure 5). Meanwhile, compared with the MC group, HPM significantly influenced the expression of 26 miRNAs in CD rats' colons (*P* < 0.05), including 9 downregulated miRNAs and 17 upregulated miRNAs (Figure 6).

### 3.4. Regulating Effects of HPM on Differentially Expressed miRNAs in CD Rats' Colons

**Figure 7:**
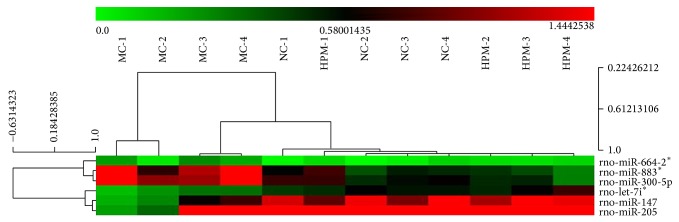
CD-related miRNAs that were regulated by HPM. Six CD-associated miRNAs that were regulated by HPM were identified by miRNA microarray. Red indicates higher than mean intensity across all samples, and green represents lower than mean intensity. *n* = 4 rats per group.

**Figure 8:**
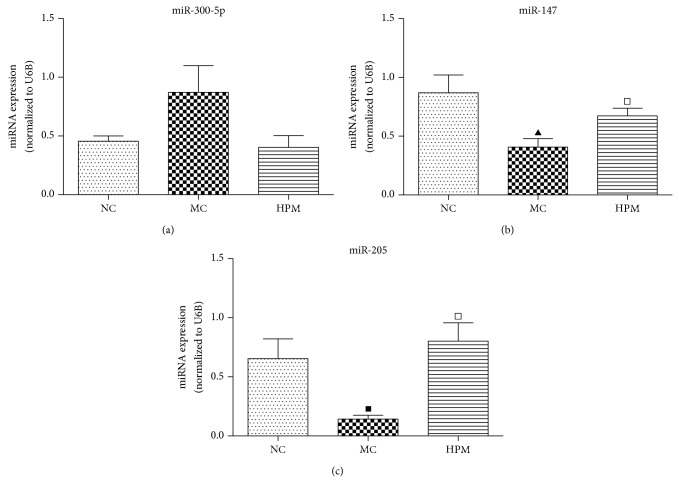
Verification of HPM's target miRNAs in CD. Three CD-associated miRNAs that could be regulated by HPM were verified by miRNA qRT-PCR. HPM treatment significantly increased the expression of miR-147 and miR-205 that were downregulated in the MC group and decreased the expression of miR-300-5p that was slightly upregulated in the MC group. Data represent means ± SEM, *n* = 6 rats per group. ^▲^ *P* < 0.01, ^■^ *P* < 0.05 versus the NC group; ^□^ *P* < 0.05 versus the MC group.

The data of all differentially expressed miRNAs were further analyzed by one-way ANOVA. As shown in Figure 7, our initial miRNA microarray profiling identified 6 CD-associated miRNAs that were regulated by HPM, of which 3 miRNAs, miR-300-5p, miR-664-2^*^, and miR-883^*^, were increased after modeling but downregulated after HPM, and the other 3 miRNAs, miR-147, miR-205, and let-7i^*^, were inhibited after modeling while being upregulated after HPM. To further verify the results from miRNA microarray analysis, 3 differentially expressed miRNAs (miR-300-5p, miR-147, and miR-205) were chosen to be tested by qRT-PCR in additional frozen colons of all groups. The U6B was used as the endogenous control, and the relative expression levels of the studied miRNAs were calculated using 2^−ΔΔCt  ^. As demonstrated in Figure 8, miRNA qRT-PCR confirmed that miR-147 and miR-205 were significantly downregulated in the MC group compared with the NC group (*P* < 0.01, *P* < 0.05, resp.); compared with the MC group, HPM significantly upregulated their expression (*P* < 0.05), the fold changes of which were 2.64 and 3.72, respectively. However, the expression of miR-300-5p only showed a tendency to increase in the MC group and to decrease in the HPM group, but without a statistical significance (*P* > 0.05). These validation results were basically consistent with the miRNA microarray results.

### 3.5. Prediction of miR-147 and miR-205 Target Genes in Rats

The target genes of miR-147 and miR-205 were predicted using online query facility TargetScan 6.2. The potential target genes of miR-147 are* BDNF*,* ZNF148*,* ZNF518B*, and so forth, and miR-205 can target hundreds of genes, like* LRP1*,* LRRK2*,* SerpinB2*,* SMAD4*,* ErbB4*,* MARCKS*,* ADAMTS9*,* MMD*,* IL-1R1*, and so forth. These potential target genes are widely involved in the regulation of many inflammatory signal pathways, such as TLR, NF-*κ*B, p38 MAPK, and Erk, and play a certain role in suppressing inflammation or proinflammation.

Researches [26, 27] have shown that the overexpression of* ErbB4* in intestinal mucosa of CD patients and CD mice may inhibit epithelial cell apoptosis induced by TNF-*α* and NF-*κ*B and ameliorate colonic tissue damage.* LRRK2* is involved in innate immune activities, and the loss of its function may cause a significant deficit in monocyte autophagy [28, 29], which is closely related to the onset of CD and colitis [30].* LRRK2* knock-out animals also demonstrate exacerbated colonic inflammation in an experimental model of colitis [31]. However, in mice models of Parkinson's disease,* LRRK2* can promote the expression of inflammatory factors in microglia through TLR4 signal pathway, thereby playing a role in proinflammation [32, 33].* LRP1*,* SerpinB2*,* SMAD4*, and* MARCKS* all can inhibit inflammatory gene transcription and expression of inflammatory cytokines (TNF-*α*, IL-1*β*, etc.), of which* LRP1*,* SerpinB2*, and* SMAD4* also inhibit NF-*κ*B and p38 MAPK signal pathways which are mediated by TNF-*α* or TLR4, thereby indirectly inhibiting the expression of inflammatory cytokines and playing a role in suppressing inflammation [27, 34–37]. In neuronal cells,* BDNF* inhibits neuroinflammation and protects nerve by downregulating the expression of inflammatory cytokines [38]. Some inflammatory cytokines such as TNF-*α* and IL-1*β* can also upregulate the expression of* BDNF* by activating NF-*κ*B so as to inhibit neuroinflammation [39, 40]. LPS stimulation increases the expression of* MMD* in macrophages and then upregulates the expression of downstream TNF-*α* and NO by phosphorylating Erk and Akt; IL-1*β* stimulation may upregulate the expression of* ADAMTS9* in chondrocytes by NBE. Thus, both* ADAMTS9* and* MMD* possess proinflammatory effects [41, 42].

## 4. Discussion

miRNAs are involved in many biological processes, including tissue morphogenesis, major signal pathways, and cellular processes like apoptosis, differentiation, and proliferation [11, 12]. They have been widely researched in various human diseases such as cancers and chronic inflammatory and autoimmune diseases [13, 14]. There is accumulating evidence that miRNAs play a significant role in the pathogenesis of IBD (including CD and UC). They may be the potential diagnostic indicators for IBD, and interventions on miRNAs may also be an effective way in IBD treatment. In samples of endoscopic pinch biopsies, the expressions of miR-23b, -106, -191, -196, -19b, and -629 have significant changes in colonic mucosa of active Crohn's colitis patients, whereas miR-16, -21, -223, and -594 have been identified to be overexpressed in chronic active ileal CD, which indicates that region-specific IBD-associated miRNAs are involved in diverse pathways responsible for the pathogenesis of different IBD subtypes [1, 43]. Zahm et al. [44] have identified 11 pediatric CD-associated serum miRNAs with encouraging diagnostic potential. It has also been demonstrated that [45], in inflammatory intestinal mucosa of CD patients and experimental colitis mice, the overexpression of CXCL12*β* may promote immune cell infiltration and induce the morbidity of IBD in human and mice; intracolonic administration of pre-miR-141 may inhibit colonic CXCL12*β* expression in mice and block immune cells trafficking into the inflamed sites, thereby significantly alleviating intestinal inflammation, which represents a promising approach that may be valuable for the treatment of CD. In addition, as up to 10% of IBD patients with colonic inflammation cannot be clearly classified into either group and are given the diagnosis of indeterminate IBD [2], some researchers have explored the value of miRNA in distinguishing UC and CD based on miRNA-related studies. miR-19b, -106a, and -629 have been confirmed differentially expressed in colon tissues of endoscopic pinch biopsies between UC and CD patients [15]. Compared with healthy controls, 11 miRNAs (such as miR-16, -23a, and -29a) are expressed at significantly higher levels in peripheral blood of CD patients, of which, the high expression of miR-16 and -106a was also confirmed by Zahm's research. However, only 3 miRNAs are significantly upregulated in blood samples of UC subjects, of which miR-155 is the most highly expressed [46]. Taken together, as crucial factors in the onset and/or relapse of mucosal inflammation in IBD patients, miRNAs may not only contribute to the susceptibility of IBD, but also have the potential to become new diagnostic biomarkers and therapeutic targets in IBD [16].

Our study has screened the miRNA expression profiling in colons of experimental CD rats and further verified CD-related miRNAs that can be regulated by HPM. The relevance between miRNAs and inflammatory signal pathway was also explored. The miRNA microarray analysis showed that 40 miRNAs were differentially expressed in CD rats' colons, with 28 miRNAs (miR-300-5p, miR-664-2^*^, miR-883^*^, miR-134^*^, miR-361^*^, miR-466d, miR-3596c, miR-23a^*^, miR-34c^*^, miR-466c^*^, miR-196c^*^, miR-3596d, miR-3594-3p, miR-935, miR-466b-2^*^, miR-3572, miR-138-1^*^, miR-465^*^, miR-466c, miR-185^*^, miR-505^*^, miR-206, miR-487b^*^, miR-877, miR-181a-2^*^, miR-2985, miR-30b-3p, and miR-103-1^*^) significantly overexpressed and 12 (let-7i^*^, miR-147, miR-205, miR-29a, miR-378, miR-133a, miR-196b, miR-200a, miR-200b, miR-204, miR-30c, and miR-708) significantly underexpressed, compared with the NC rats. Meanwhile, HPM regulated 26 miRNAs in colon tissues compared with the MC group, 9 (miR-300-5p, miR-664-2^*^, miR-883^*^, miR-361^*^, miR-134^*^, miR-466d, miR-3596c, miR-299, and miR-433^*^) of which were downregulated and 17 (let-7i^*^, miR-147, miR-205, miR-29a, miR-378, miR-499, miR-29a^*^, miR-101a, miR-139-5p, miR-194, miR-145^*^, miR-423, miR-138, miR-434^*^, miR-29c, miR-9, and miR-196b^*^) upregulated. Among all the 40 differentially expressed miRNAs in the MC group, only 6 CD-associated miRNAs were influenced by HPM. Since 3 of them (miR-664-2^*^, miR-883^*^, and let-7i^*^) have no predicted targets on TargetScan (http://www.targetscan.org/) and miRBase (http://www.mirbase.org/), only miR-300-5p, miR-147, and miR-205 were chosen to be further validated by miRNA qRT-PCR. Finally we found that downregulated miR-147 and miR-205 in CD rats' colons were both significantly upregulated by HPM, with 2.64- and 3.72-fold increase, respectively. miR-300-5p showed a trend towards upregulation in the MC group and downregulation in the HPM group, but without statistical significances (*P* > 0.05), so it was excluded from further study.

Previous researches [47] have demonstrated that, in murine macrophages stimulated by TLR ligands, NF-*κ*B and/or IRF3 promote the activation of miR-147, and the activated miR-147 negatively regulates the excessive inflammatory responses in macrophages. Further study revealed that miR-147 knock-out could increase inflammatory cytokine expression and transfection of miR-147 mimics into macrophages may significantly decrease LPS-induced TNF-*α* and IL-6 production as well as the expression of several other LPS-induced genes and miR-147 itself. Although the predicted target genes of miR-147 have not been demonstrated to be directly related to TLR signal pathway, and miR-147 cannot change the phosphorylation of I*κ*B*α* and NF-*κ*B p65, this study has confirmed miR-147 activation induced by TLR stimulation may negatively regulate the excessive inflammatory responses in macrophages, and its mechanism needs further study. In addition, researches have also demonstrated that miR-147 may inhibit the expression of proinflammatory cytokines in peripheral blood cells of dengue-infected patients [48].

For now, most researches related to miR-147 and miR-205 are associated with cancers. miR-147 has been demonstrated to be a potential tumor suppressor, potential for diagnosing and treating tumors. It may inhibit the onset, development, and metastasis of tumor by reversing EMT, inhibiting cell cycle and cell proliferation, and by increasing the sensitivity of tumor patients to chemotherapeutic drugs [49, 50]. miR-205 can not only suppress tumors, but also play a role in promoting tumor progression. In breast cancer, prostate cancer, and other tumor diseases, miR-205 may inhibit tumor cell proliferation, invasion, and tumor growth by reversing EMT and downregulating the expression of ZEB1/2 [51–53], and it may also increase the sensitivity of tumor cells to chemotherapeutic drugs [54], thereby playing a role in tumor suppression. As a tumor-promoting factor, miR-205 may promote the onset, development, and metastasis of tumors and increase tolerance of tumor cells to radiotherapy and chemotherapy by targeting and inhibiting tumor-suppressing genes such as PTEN in tumors like non-small cell lung cancer and endometrial cancer, resulting in a decreased survival rate in the patients [55, 56].

It has been demonstrated that miR-147 overlaps with* NMES1* transcript and may be upregulated by LPS stimulation in both murine and human macrophages, suggesting that miR-147 expression in inflammatory cells is conserved across species [47]. The expression of miR-205 also showed the similar trends in murine and human melanoma cells after curcumin treatment [57]. Moreover, the sequences of miR-147 and miR-205 are also highly homogenous in rats and human (http://www.mirbase.org/). Therefore, they may have similar expression profiling and biological functions across these two species, and further study is needed in the future.

Retrospective studies have shown that not only CD patients suffer a high risk of colorectal cancer and lymphoma, but also many of their parenteral organs, such as liver, kidney, and testicles, are at increased risks of tumors [58]. In our study, the expression of miR-147 and miR-205 was significantly decreased in colons of experimental CD rats, indicating that their downregulation was closely related to CD pathogenesis, and might increase the possibility of experimental CD rats in developing colon cancer and other tumor diseases. HPM could significantly upregulate the expression of miR-147 and miR-205 in CD rats' colons, initially and indirectly affirming that HPM should be potential in preventing pathogenesis of tumor diseases.

In summary, our research has found that HPM at Tianshu and Qihai of experimental CD rats can not only significantly improve the histopathological damage of CD rats' colons, but also target and upregulate miR-147 and miR-205 in CD rats' colons. The mechanism of HPM in treating CD may be through upregulating the expression of miR-147 and/or miR-205 that are abnormally downregulated in CD rats' colons to further regulate some of their target genes, thereby indirectly inhibiting the inflammatory signal pathways mediated by TLR, NF-*κ*B, and so forth and decreasing the production of downstream inflammatory cytokines such as TNF-*α* and IL-1*β* [19], so as to alleviate intestinal inflammation in CD. Meanwhile, HPM may also reduce the risk of tumor diseases such as colon cancer. Although miR-155 and miR-126 have been demonstrated to promote intestinal inflammation in IBD by inhibiting the expression of NF-*κ*B inhibitor I*κ*B*α* [59, 60], they are not the miRNA targets in HPM treatment on Tianshu and Qihai of experimental CD rats, indicating that HPM has specificity in terms of gene regulation. This research provides a new idea to clarify the mechanism of HPM in treating experimental CD and provides an initial theoretical basis of HPM in preventing the pathogenesis of tumor diseases, which is worthy of further study. However, our study still has some regrets. For example, the mechanisms of HPM in regulating the expression of miRNAs and miRNAs in negatively regulating inflammatory signal pathways have not been fully elucidated and need further study. In addition, CD clinical studies cannot be fully represented by animal experiments. Thus, in future researches, we need to carry out large-scale, randomized, double-blind, and controlled clinical trials based on miRNAs of colonic mucosa and blood of CD patients and design a long-term follow-up in CD patients, in order to determine the important miRNA expression profiling in the pathogenesis of CD, and explore the detailed mechanism of HPM in treating CD and preventing tumors at the genetic and molecular levels.

## Figures and Tables

**Table 1:** The sequences of RT primers.

	Gene	Stem-loop RT primers
Rat	U6B	CTCAACTGGTGTCGTGGAGTCGGCAATTCAGTTGAGAAAATATG
Rat	miR-300-5p	CTCAACTGGTGTCGTGGAGTCGGCAATTCAGTTGAGACAAAGGA
Rat	miR-147	CTCAACTGGTGTCGTGGAGTCGGCAATTCAGTTGAGTAGCAGAA
Rat	miR-205	CTCAACTGGTGTCGTGGAGTCGGCAATTCAGTTGAGACAGACTC

**Table 2:** The sequences of PCR primers.

	Gene	Forward primers	Reverse primers
Rat	U6B	5′-CAAATTCGTGAAGCGTT-3′	5′-GGAGTCGGCAATTCAG-3′
Rat	miR-300-5p	5′-TGAAGAGAGGTTATCCTTT-3′	5′-CTGGTGTCGTGGAGTC-3′
Rat	miR-147	5′-GTGTGCGGAAATGCTT-3′	5′-TCAACTGGTGTCGTGG-3′
Rat	miR-205	5′-TCATTCCACCGGAGTC-3′	5′-CAACTGGTGTCGTGGAG-3′
